# Ticks infesting domestic dogs in the UK: a large-scale surveillance programme

**DOI:** 10.1186/s13071-016-1673-4

**Published:** 2016-07-07

**Authors:** Swaid Abdullah, Chris Helps, Severine Tasker, Hannah Newbury, Richard Wall

**Affiliations:** Veterinary Parasitology and Ecology Group, School of Biological Sciences, Life Sciences Building, University of Bristol, Bristol, UK; Molecular Diagnostic Unit, Langford Veterinary Services and School of Veterinary Sciences, University of Bristol, Bristol, UK; MSD Animal Health, Walton Manor, Walton, Milton Keynes, UK

**Keywords:** Tick, Surveillance, Relative risk, Vector, *Ixodes*, *Dermacentor*, *Rhipicephalus*

## Abstract

**Background:**

Recent changes in the distribution of tick vectors and the incidence of tick-borne disease, driven variously by factors such as climate change, habitat modification, increasing host abundance and the increased movement of people and animals, highlight the importance of ongoing, active surveillance. This paper documents the results of a large-scale survey of tick abundance on dogs presented to veterinary practices in the UK, using a participatory approach that allows relatively cost- and time-effective extensive data collection.

**Methods:**

Over a period of 16 weeks (April–July 2015), 1094 veterinary practices were recruited to monitor tick attachment to dogs and provided with a tick collection and submission protocol. Recruitment was encouraged through a national publicity and communication initiative. Participating practices were asked to select five dogs at random each week and undertake a thorough, standardized examination of each dog for ticks. The clinical history and any ticks were then sent to the investigators for identification.

**Results:**

A total of 12,000 and 96 dogs were examined and 6555 tick samples from infested dogs were received. *Ixodes ricinus* (Linnaeus) was identified on 5265 dogs (89 %), *Ixodes hexagonus* Leach on 577 (9.8 %) and *Ixodes canisuga* Johnston on 46 (0.8 %). Ten dogs had *Dermacentor reticulatus* (Fabricius), one had *Dermacentor variabilis* (Say), three had *Haemaphysalis punctata* Canesteini & Fanzago and 13 had *Rhipicephalus sanguineus* Latreille. 640 ticks were too damaged for identification. All the *R. sanguineus* and the single *D. variabilis* were on dogs with a recent history of travel outside the UK. The overall prevalence of tick attachment was 30 % (range 28–32 %). The relatively high prevalence recorded is likely to have been inflated by the method of participant recruitment.

**Conclusion:**

The data presented provide a comprehensive spatial understanding of tick distribution and species abundance in the UK against which future changes can be compared. Relative prevalence maps show the highest rates in Scotland and south west England providing a valuable guide to tick-bite risk in the UK.

## Background

Ticks are globally important arthropod vectors of disease that transmit an extensive range of viral, bacterial and protozoan pathogens to vertebrate hosts [1]. Tick survival, phenology and biting activity is highly dependent on environmental conditions [2] and is highly responsive to changes in factors such as climate and habitat modification. Long-term increases in the abundance of ticks, such as *Ixodes ricinus* (Linneaus)*,* have been recorded in temperate habitats over recent decades [3], along with evidence of altitudinal and latitudinal expansion in central and northern Europe [4–8]. Data collected by questionnaire from 20 districts in Sweden showed a significant increase in tick abundance in west-central regions where previously ticks had been rare. Blanket dragging in 54 regions along the perceived latitudinal boundary for ticks supported this observation [9, 10]. Similarly, in the UK, over recent decades there has been an estimated 17 % expansion in the distribution of *I. ricinus* and an increase in abundance at 73 % of locations surveyed [11]. The tick *Dermacentor reticulatus* (Fabricius), an important vector of canine babesiosis in Europe, is also believed to have extended its distribution and populations have become established in Poland [12], Belgium [13], Germany [14], the Netherlands [15] and in southern England [16, 17]. Changes in tick-borne disease prevalence are also reported, in association with the changes in vector distribution [18, 19].

Climate change is considered likely to be responsibe in part for these observed changes [20] and further climate-related impacts might be expected if predicted increases in global temperatures of up to 4.8 °C in the next hundred years occur [21]. Longer summer seasons, with a warmer and wetter spring or autumn, might be expected to promote higher tick challenge and longer exposure. Tick mortality may be lower given milder winters, but higher in hotter drier summers. In addition, vector potential may be enhanced by biological changes stimulated by temperature, as is reported in other arthropod vectors [22]. However, ticks may also adapt their seasonal activity and some species may aestivate during very hot conditions and perhaps adopt a more bimodal pattern of activity, pushing the period of feeding to earlier and later in the year. Nevertheless, along with climate, changes in habitat management, land use by people and animals, host movement patterns and changes in host abundance, particularly deer, may also be equally important in explaining changes in patterns of tick abundance and activity [23].

A central problem associated with understanding changing arthropod-borne disease patterns is that systematic surveillance in animals is not routinely undertaken [24]. The cost and complexity of this monitoring is high because vector and pathogen prevalence are often relatively low and large samples sizes are required for meaningful results. Surveillance programmes often rely on passive reporting, which may render them subject to significant levels of spatial and temporal bias. Nevertheless, systematic surveillance is essential to allow detection of changes in the distribution of arthropod vectors and arthropod-borne diseases, particularly because subtle changes at the early stages of an epidemic curve are hard to spot. Routine surveillance is also needed to allow informed risk analysis and the evaluation of the potential spread to new areas or the new introduction of exotic species or diseases [24–26]. This necessitates clear and exhaustive knowledge of the distribution of arthropod vectors and associated vector-borne diseases in different areas [1].

The aim of the work described here therefore was to undertake a national survey of tick abundance on dogs presented to veterinary practices in the UK and to provide a comprehensive spatial understanding of the distribution and species abundance against which future changes could be compared. This study also aimed to evaluate an approach to large-scale surveillance that allows relatively cost- and time-effective extensive data collection.

## Methods

Veterinary practices from throughout the UK were recruited largely through an intensive media and communication initiative, designed to raise awareness of ticks and tick-borne disease amongst veterinarians and the general public. The campaign, launched in March 2015, used an intense period of radio, television, print and social media to highlight the role of ticks as vectors of canine disease in the UK. Interested veterinary practices were then able to register their interest by e-mailing a contact name and their practice details. Once enrolled, they were sent a pack which contained 40 questionnaires, stamped addressed envelopes, a tick removal hook, specimen tubes and protocols. The protocol asked registered veterinary practices to examine five different dogs for ticks each week, for a total of 8 weeks, using a specified standard grooming procedure and then complete a questionnaire relating to the clinical and travel history of each dog. Veterinarians were asked to ensure that the dogs selected for inspection were a random cross-section of animals brought into the surgery for routine procedures such as vaccination and were not those known to be carrying ticks when selected, but no formal randomisation procedure was included in the protocol.

The examination protocol for included dogs required first checking the head for ticks. Special attention was given to the ears, carefully checking the pinnae and inside the external ear canal. The dog was then checked on the neck and chest area, legs, armpits and interdigital spaces. After that the dog’s hair, from head to tail, was checked manually using sufficient pressure to detect small lumps. Finally, a louse or flea comb was used to part the hair along the length of the body. The examination was estimated to take an average of 5 min per dog. All attached ticks were removed using a tick hook or forceps, placed in a tube labelled with the dog’s name and inspection date and stored in a freezer at -20 °C. Each week stored ticks along with the completed questionnaires were posted to the investigators. The instructions sent to each registered veterinary practice stressed the importance of completing and sending questionnaires for dogs that were found not to have ticks, to allow a true prevalence figure to be calculated.

Each tick sample received by the investigators was given a unique identification number and placed in freezer at -20 °C pending analysis. Subsequently, ticks were identified to species, life-cycle stage and sex using a range of keys [27–29] and a sub-sample of identifications cross-checked by an independent investigator. The questionnaire data were entered into an Excel (Microsoft) worksheet.

The history and sex, breed and age of each dog were recorded. To determine any effect of sex on tick attachment, four categories were considered: male, female, male neutered and female neutered. For the effect of breed, the dogs were categorised following Kennel Club classifications: gundogs, hounds, pastoral, terriers, toy, utility and working, plus mongrels and crossbreds. To assess any effects of age, dogs were divided into five classes: less than 1 year-of-age, 1 to 3, 4 to 6, 7 to 10 and any of greater than 10 years-of-age. Each dog’s travel history and any history of visits to kennels were also recorded. The data were subjected to binary logistic regression using SPSS (version 23). The distribution of the participating veterinary practices, dogs and various tick species were mapped using QGIS (version 2.8.1) using the practice or owner’s postcodes.

## Results

**Fig. 1:**
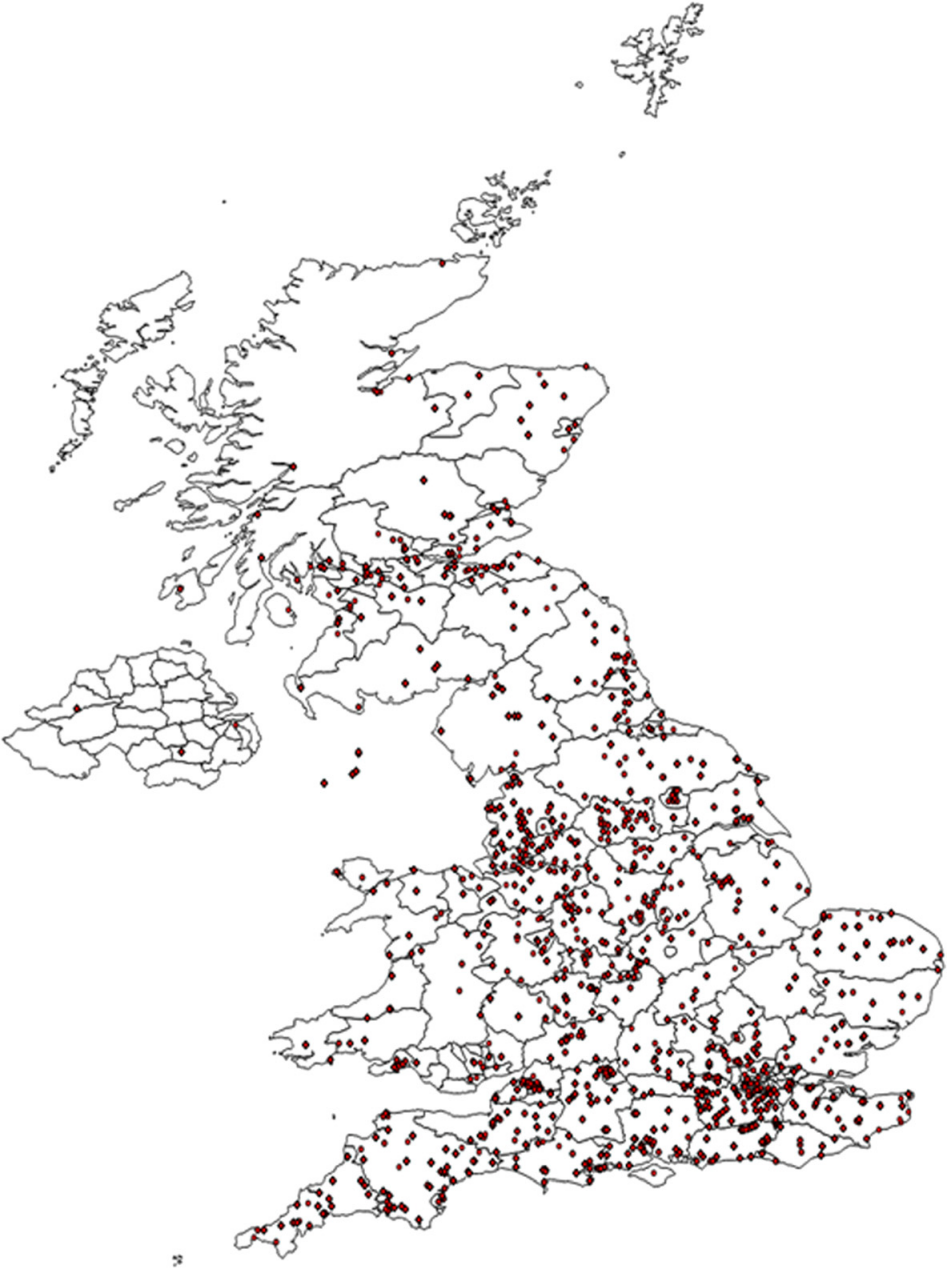
Distribution of the 1094 veterinary practices that participated in the UK survey for ticks on dogs over 16 weeks (March to July) in 2015

A total of 1000 and 94 veterinary practices from across the UK participated in the 16 week study (Fig. 1); in this time 12,096 dogs were examined for each of which a completed questionnaires was submitted. 6555 tick samples were also submitted, of which 5915 were identified, while 640 were too damaged to allow identification. The median number of ticks per sample was 1, although the maximum number reported on an individual dog was about 200. Of the tick samples received, 98.7 % were adults alone, 0.83 % were nymphs alone and 0.11 % were larvae alone. All the remaining samples were mixed stage. The nymphs and adults were identified to species, the very small numbers of larvae received were not identified. Almost all ticks were semi- or fully-engorged.

### Species abundance and distributions

**Fig. 2:**
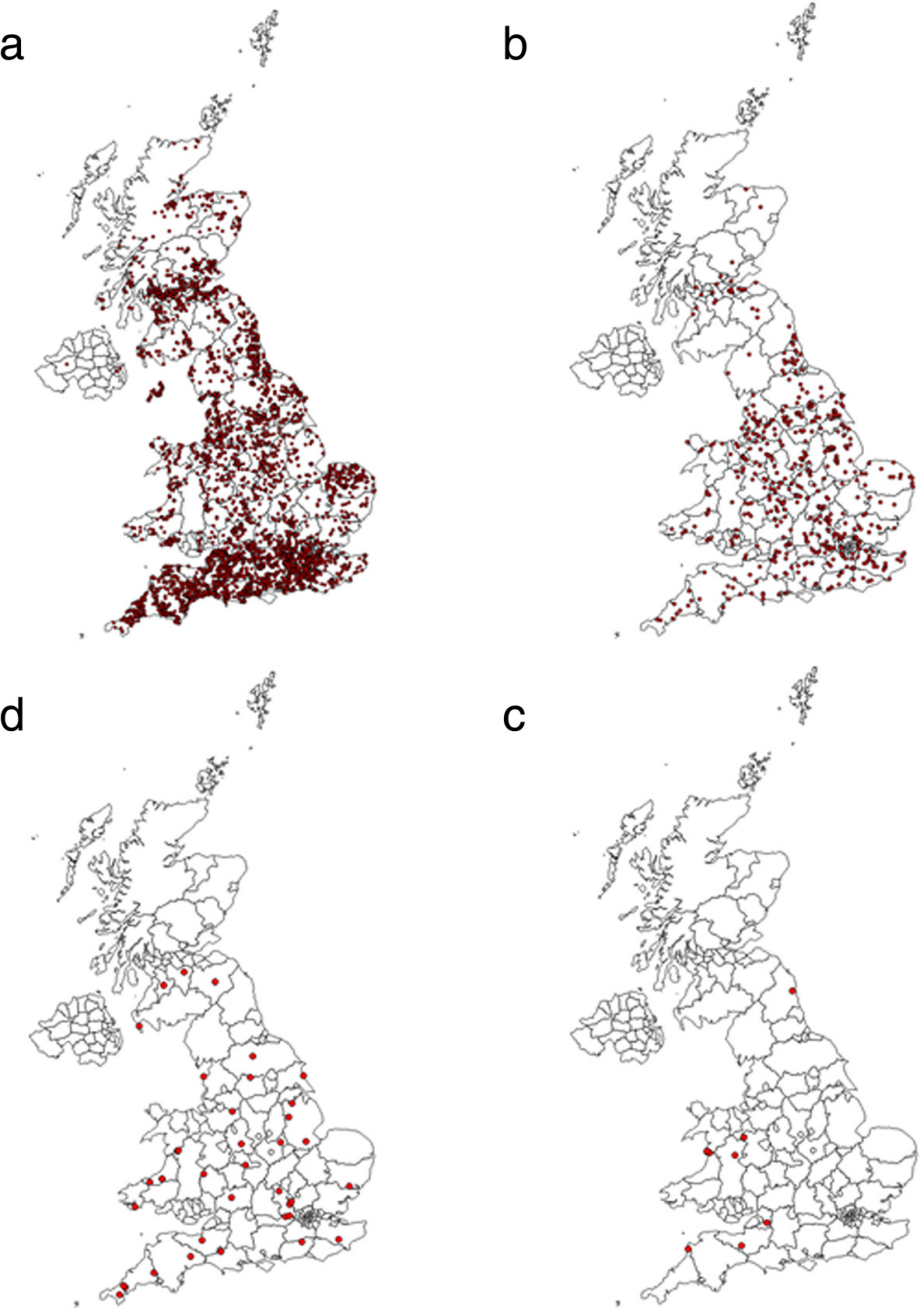
Distributions of the ticks *Ixodes ricinus* (**a**), *Ixodes hexagonus* (**b**), *Ixodes canisuga* (**c**) and *Dermacentor reticulatus* (**d**) in samples submitted by the veterinary practices in the UK survey

Amongst dogs that had not travelled outside the UK, 89.2 % of those that had ticks were infected by *I. ricinus* and 9.8 % by *Ixodes hexagonus* Leach (Table 1). Both species were widely distributed throughout the UK (Fig. 2a, b). Smaller numbers of *Ixodes canisuga* Johnston were recorded, less than 0.78 % of the dogs were infected by this species, and these tended to have a more southerly distribution in England and Wales with none submitted from the northern half of Scotland (Fig. 2c). Ten dogs were found to be infected with *D. reticulatus*, largely from populations in western Wales and south-west England (Fig. 2d). The one case recorded in north-east England, was from a dog known to have travelled to Wales in the previous week. Three dogs were infected by *Haemaphysalis punctata* Canestrini & Fanzago; all were from one specific location in south east England (not plotted). Five dogs with mixed species infestations were detected, in three of these cases *I. ricinus* was found along with *I. hexagonus*, whereas in other two *I. ricinus* was found with *I. canisuga* and *D. reticulatus*.

**Table 1:** The number and percentage of dogs that had not travelled outside the UK in the previous 2 weeks, infested by each species of tick as submitted by veterinary practices that participated in the UK survey

Tick species	Number of dogs	Percentage
*Ixodes ricinus*	5236	89.2
*Ixodes hexagonus*	577	9.8
*Ixodes canisuga*	46	0.78
*Haemaphysalis punctata*	3	0.05
*Dermacentor reticulatus*	10	0.17
Total number of dogs infested by endemic ticks	5872

Fifty-six dogs had travelled outside the UK in the previous 2 weeks (Table 2); 29 were infected with *I. ricinus*. Thirteen were infected by *Rhipicephalus sanguineus* Latrille all of which were found on animals that had entered from the Mediterranean region (predominantly Cyprus and Spain). One dog with *Dermacentor variabilis* (Say) was detected, on an animal imported from the USA.

**Table 2:** Amongst the samples submitted by veterinary practices, 56 dogs had travelled outside the UK in the previous 2 weeks, 43 of which were infested by one of three species of tick

Tick species	Number of travelled dogs infested (%)
*Ixodes ricinus*	29 (67.4)
*Dermacentor variabilis*	1 (2.3)
*Rhipicephalus sanguineus*	13 (30.2)
Total number with ticks	43

### Host associations and risk factors

Logistic regression was used to consider the effects of various risk factors on the likelihood that dogs may get bitten by ticks. This analysis showed that breed, neutered status and age significantly predicted the likelihood of dogs having ticks, but visits to kennels and exposure to different habitats did not (Table 3). Pastoral and Gundogs were the breeds most likely to have ticks (*R*^2^ = 0.037, *df*_9, 17_, *P* < 0.001); neutered male and female dogs were at lower risk of tick infestation than male or female unneutered dogs (*R*^2^ = 0.037, *df*_3, 17_, *P* < 0.001). All age groups were significantly more likely to have ticks than dogs of 1 year-old or below (*R*^2^ = 0.037, *df*_5, 17_, *P* < 0.001).

**Table 3:** Significance, odds ratios and 95 % confidence intervals (CIs) of the logistic regression between presence and absence of ticks and an array of significant tick risk factors

	Significance	Odds ratio	95 % CI
Breed type			
Pastoral	0.009	1.809	1.162–2.817
Gundogs	0.026	1.619	1.060–2.471
Dog sex			
Male neutered	< 0.0001	0.638	0.553–0.736
Female neutered	< 0.0001	0.570	0.494–0.659
Dog age			
1 to 3 years	< 0.0001	1.400	1.184–1.655
3 to 6 years	0.001	1.331	1.128–1.571
6 to 10 years	0.036	1.202	1.012–1.427
Above 10 years	0.014	1.288	1.052–1.577

Hosmer-Lemeshow test: *χ* ^2^ = 2.117, *df* = 8, *P* = 0.977

### Prevalence of tick attachment

**Fig. 3:**
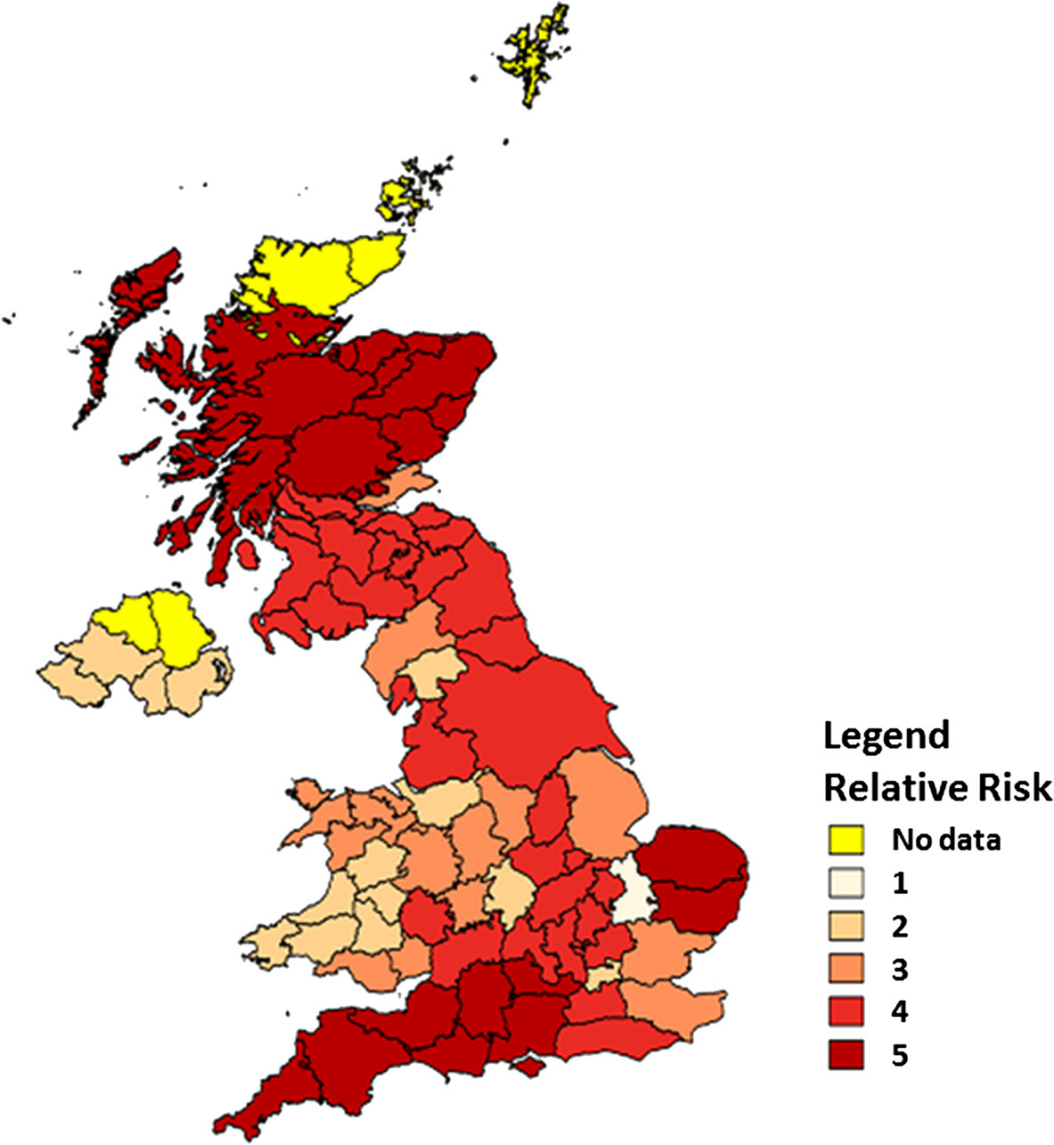
The relative risk of tick attachment on dogs on a scale of 1 to 5, based on the prevalence of ticks found in different regions of the UK in samples submitted by the veterinary practices in the UK survey

Prevalence estimation requires random selection of dogs without bias towards dogs known to have attached ticks. However, while many participating veterinary practices, as expected, sent more negative questionnaire reports than positive, some veterinary practices sent questionnaire reports only from infested dogs. It is therefore likely that these practices misunderstood the study protocol and only submitted reports when ticks were found. All data submitted from practices that sent only positive samples from any single week in which it participated in the survey were not included in the prevalence analysis. Data from practices that submitted reports from three or fewer dogs any week were also removed, since it was considered unlikely that any practice would see fewer than three dogs in a week. This removal of inspection records from practices where miss- or over-reporting was suspected, resulted in the removal of 4994 dogs. Following this, the total number of dog records remaining was 7102 and of these 2182 of the dogs had ticks. The prevalence of ticks on dogs over the entire 16-week period was thus 30.7 % (95 % exact binomial confidence interval ± 0.011) (Table 4). Calculation of the prevalence over each four-week period of the study showed little variation, with a range of 28–32 % (Table 4). The prevalence data were mapped by county to give a visual indication of geographical differences (Fig. 3). Where there were small sample sizes from any one county, counties were merged to give a minimum sample of 200 cases per reporting area and a pooled prevalence was plotted. Prevalence was then mapped on a relative scale of 1 to 5. The data show that the highest prevalence of tick infestation are in south west England, East Anglia and Scotland, but are also high throughout most of central and northern England.

**Table 4:** The number of dogs inspected and the number found to have at least one tick attached in each 4 week period of the 16 week study, in samples submitted by veterinary practices in the UK survey

Time period (weeks)	Number of dogs	Number of tick infested dogs	Prevalence (%)	95 % confidence interval
1–4	287	81	28.2	0.052
5–8	1784	503	28.2	0.021
9–12	2938	959	32.6	0.017
13–16	2093	639	30.5	0.019
1–16	7102	2182	30.7	0.011

The estimated percentage prevalence with exact binomial 95 % confidence intervals are also presented

## Discussion

The three tick species most commonly encountered on humans, livestock and companion animals in the UK are *Ixodes ricinus*, *I. hexagonus* and *I. canisuga*; of these, *I. ricinus* is the most widely distributed and poses the greatest biting risk [16]. This species is the known principal vector for Lyme borreliosis and *Anaplasma*, louping ill virus (LIV) and various species of *Babesia* [2, 6, 30]. In mainland Europe *I. ricinus* also acts as a vector of tick-borne encephalitis (TBE) in humans and *Ehrlichia canis*; the latter has been recorded recently for the first time in a non-travelled dog in the UK [31]. In a previous study, based on 56 veterinary practices and 280 animals in the UK, 52 % of dogs and cats with ticks carried *I. ricinus*, 39 % carried *I. hexagonus*, and 11 % were infested with *I. canisuga* [32]. More recently, a study involving 180 veterinary practices found that 72.1 % of tick infestations on domestic dogs were due to *I. ricinus*, 22 % due to *I. hexagonus* and 6 % due to *I. canisuga* [17]. In the present study, data collected from over 1000 veterinary practices and 12,000 dogs showed that the proportion of *I. ricinus* was considerably higher than in earlier studies at 89 %, while *I. hexagonus* was the second most abundant species (10 %) and *I. canisuga* was relatively rare (1 %). Despite differences from study to study, the pattern of abundance of the three species appears relatively consistent; variation may be due to differences in time of year, geographic focus or the sample population examined.

The abundance of each tick species is strongly determined by differences in climate, host availability and vegetation cover, which affects microclimate. *Ixodes ricinus* is most commonly associated with woodland and moorland habitats, although high densities may also be found in urban recreational spaces [33] as with *I. hexagonus* [32]. In the present study, both species were found throughout the UK as far north as Scotland. In contrast, *I. canisuga* has been reported as being more commonly found in boarding kennel environments [27], although infestation by *I. canisuga* was seen here in dogs that had no previous exposure to kennels, as has been reported previously [32]. Hence this species evidently lives in association with wildlife hosts, such as mustelids [27]. As seen in previous studies [17], *I. canisuga* appeared to have a more strongly southerly distribution in the current study.

Risk factors associated with tick attachment are highly inconsistent between studies. Dog breed, sex, age and neutered status were not found to be significant predictors of tick infestation [34]. Earlier work [17] found that hound, toy and utility breeds and neutered dogs had a lower probability of tick attachment, but reported no significant effect of sex. In the current study, older dogs were more likely to have ticks than dogs of 1 year-of-age or below, pastoral and gundogs were at higher risk than other breeds while neutered dogs were at lower risk than unneutered dogs. No effect of sex was detected. It is possible that the highly variable results between studies are due to the fact that tick attachment rate has been shown to be most strongly correlated with exposure rather than any dog physiological or phenotypic characteristics, as demonstrated by Jennet et al. [34]. Surprisingly, dogs that were restricted to urban habitats were no less likely to have ticks that dogs exposed to more rural habitats. This corresponds with the growing number of reports of high numbers of ticks in urban environments [32, 33]. Almost all the ticks found were adults; only 76 were immatures. It is relatively likely that dogs are bitten by a greater proportion of immatures than is apparent from the data presented here, but their absence in the samples received strongly suggests that this life-cycle stage is undetected in clinical examination.

The results of this study show that a large-scale, cost-effective national tick prevalence assessment can be conducted using voluntary enrolment. Publicity and media interest were pivotal to the approach, generating enthusiasm amongst the participating veterinary surgeons. However, there were evident limitations; veterinarians who signed up agreed to inspect a given number of dogs per week and these were to be a random selection of otherwise healthy animals brought to the surgery. In the study reported by Smith et al. [17] which used a similar protocol, 60 veterinary practices participated in tick collection at any one time and incoming samples were monitored closely and veterinary practices were contacted individually by telephone when unexpected patterns were detected to ensure that the protocols were rigorously followed. Given the number of veterinary practices that participated in the current study, this approach could not be adopted and more than half of participating practices sent too few or only positive samples for at least 1 week during their participation. Furthermore, over 200 ticks were sent from cats (plus samples from humans and birds). On the other hand, the very large sample size meant that a more rigorous *post-hoc* approach could be taken to exclude specific categories of return.

After exclusion of returns where only cases from dogs positive for tick infestation were submitted, an overall attachment prevalence on dogs of 30 % was recorded over the 16-week sample period. There was little change in this prevalence figure when sub-divided into 4-week periods (range 28–32 %). This attachment rate is still relatively high; in the study by Smith et al. [30] a median frequency of infestation of 14.9 % was reported, but 19 % of veterinary practices found no ticks and 14.6 % reported that more than 50 % of the dogs inspected carried ticks. In the present study, it is likely that the veterinarians who enrolled were those with the greatest interest in ticks and tick-borne disease or were in practices with a known history of tick problems amongst their clients resulting in over-reporting. Nevertheless, if it is assumed that over-reporting has no regional bias, the data can be used on a relative scale to compare geographical differences in relative risk. Scotland, south-west England and East Anglia showed the highest regional prevalence, although prevalence was also high throughout central and northern England. The relatively low prevalence in Wales was perhaps surprising, given the vegetation and climatic requirements, particularly of *I. ricinus*.

The distribution of *D. reticulatus* is of particular current interest because it is the primary vector of canine babesiosis. Much like *I. ricinus*, *D. reticulatus* is also known to have extended its distribution northwards through mainland Europe [13, 15, 35]. Historical records show that this tick has been found in the UK for over 100 years (http://data.nbn.org.uk). However, in recent years it has become more widely established in southern England and Wales [17, 36]. There are now known to be at least four established predominantly coastal populations: west Wales, south Devon, north Devon and Essex [16, 36]. Other populations may as yet be undiscovered. Here, samples were found in Wales and south-west England, confirming established distribution pattern of this species in these areas, but no cases were submitted from the Essex population. In the UK, there have been an increasing number of cases of babesiosis in dogs that have travelled to Europe [37], with other cases probably unreported [38]. In March 2016, a cluster of cases of canine babesiosis, due to *B. canis*, was reported in Essex in non-travelled dogs confirming that this pathogen is now well established in the UK [39]. This outbreak highlights the urgent need for an improved understanding of the ecology and behaviour of the vector, and in particular, an understanding of its distribution and mechanism(s) of dispersal.

Fifty-six dogs were known to have travelled outside the UK in the 2 weeks prior to their inclusion in the study and 43 of these were found to be carrying attached ticks, predominantly *I. ricinus*. The 13 cases of the tick, *R. sanguineus*, that were detected in travelled dogs, highlight the concern regarding the import and potential establishment of this species following changes to the pet passport scheme in 2012 which had previously required dogs entering the UK to be treated against ticks [26]. This species has now been shown to have overwintered in the UK in at least two locations [26]. The data clearly emphasise the importance of appropriate treatment against ticks for dogs that are travelling and the persistent threat of introduction and establishment of non-endemic ticks and their pathogens into the UK.

## Conclusions

This study has shown how very large samples can be generated through voluntary participation of veterinary surgeries following a high profile media and communication campaign. However, despite a clear protocol for participants, this approach resulted in a prevalence of tick infestation that is considerably higher than seen in previous studies, probably as a result of over-reporting. Nevertheless, the data presented provide a comprehensive spatial understanding of tick distribution and species abundance against which future changes can be compared while the relative prevalence maps show the highest rates in Scotland and south-west England, providing a valuable guide to tick-bite risk in the UK.
